# Supplementary material for: Splice-Junction-Based Mapping of Alternative Isoforms in the Human Proteome
Source: Cell Rep. Author manuscript; Available in PMC 2020 Jan 15. (PMC6961840; doi:10.1016/j.celrep.2019.11.026)
Supplement: 2 [file NIHMS1546469-supplement-2.zip › DF1/PXD010154/Lung/Lung_26_RFWD2_QLEAQLEQIQK.pdf]

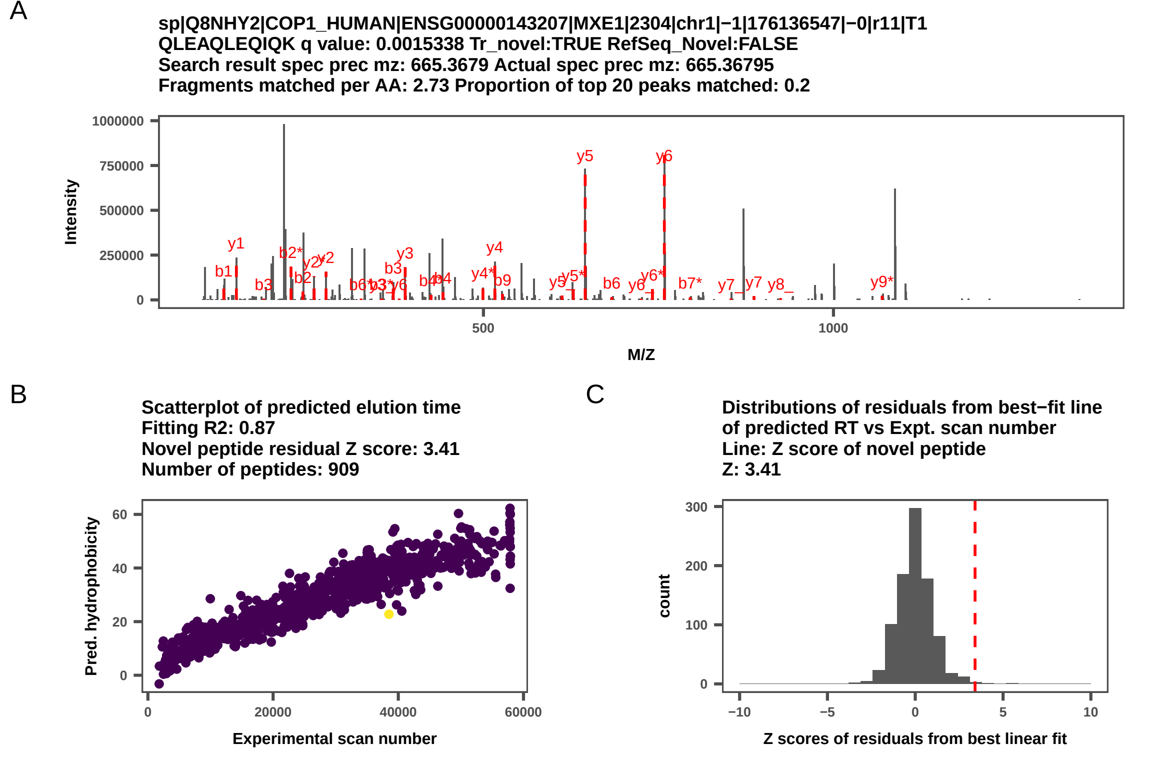
A
sp|Q8NHY2|COP1_HUMAN|ENSG00000143207|MXE1|2304|chr1|−1|176136547|−0|r11|T1
QLEAQLEQIQK q value: 0.0015338 Tr_novel:TRUE RefSeq_Novel:FALSE
Search result spec prec mz: 665.3679 Actual spec prec mz: 665.36795
Fragments matched per AA: 2.73 Proportion of top 20 peaks matched: 0.2
Intensity
1000000
750000
500000
250000
0
y1
b1
b3
b2*
b2
y2*
y2
y3
b3
y4
y4*
b9
y5
y6
b6
b7*
y7
y8
y9*
500
1000
M/Z
B
Scatterplot of predicted elution time
Fitting R2: 0.87
Novel peptide residual Z score: 3.41
Number of peptides: 909
Pred. hydrophobicity
60
40
20
0
0
20000
40000
60000
Experimental scan number
C
Distributions of residuals from best−fit line
of predicted RT vs Expt. scan number
Line: Z score of novel peptide
Z: 3.41
count
300
200
100
0
−10
−5
0
5
10
Z scores of residuals from best linear fit
